# Albuminuria and Mental Illness Risk: Results From National Health and Nutrition Examination Survey 2005–2018 and Mendelian Randomization Analyses

**DOI:** 10.1002/brb3.70545

**Published:** 2025-05-11

**Authors:** Yangyang Wang, Sen Li

**Affiliations:** ^1^ Second Medical College of Wenzhou Medical University Wenzhou China; ^2^ School of Basic Medical Sciences Wenzhou Medical University Wenzhou China

**Keywords:** albuminuria, cross‐sectional study, mental illness risk, National Health and Nutrition Examination Survey, persistent delusional disorder, schizophrenia

## Abstract

**Background:**

Recent evidence suggests a link between albuminuria and mental illness. However, whether this association is stable, and its specific mechanisms remain unclear.

**Methods:**

The cross‐sectional study utilized data from the National Health and Nutrition Examination Survey (NHANES) 2005–2018. Weighted multivariable‐adjusted logistic regression, subgroup analysis, interaction tests, and restricted cubic spline (RCS) were conducted to assess the correlation between albuminuria and the risk of mental illness (depression). Subsequently, two‐sample Mendelian randomization analyses were performed to investigate the relationship between albuminuria and various mental illnesses (anxiety disorder, persistent delusional disorder, schizophrenia, schizotypal personality disorder, panic disorder, post‐traumatic stress disorder [PTSD], obsessive‐compulsive disorder, bipolar I disorder, bipolar II disorder, depression, autism, social anxiety disorder).

**Results:**

Albuminuria was consistently found to have a significant association with the risk of depression, regardless of its classification as a continuous or outcome variable. A positive correlation was found between albuminuria and depression in different age groups, gender, race, education attainment, and those with hypertension, coronary heart disease, and diabetes. Further, there is a positive correlation between albuminuria and the occurrence of schizophrenia and persistent delusional disorder.

**Conclusion:**

There is a close association between albuminuria and mental illness, with albuminuria being a risk factor for schizophrenia and persistent delusional disorder. Further research is needed to establish the specific connections.

## Graphical abstract

Albuminuria is significantly associated with an increased risk of depression and certain mental illnesses, including schizophrenia and persistent delusional disorder, as shown by NHANES data and Mendelian randomization analysis.

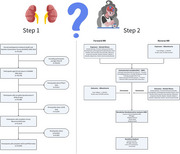

**Abbreviations**
AA: Associate of Arts
CI: confidence intervals
CKD: chronic kidney disease
GWASs: genome‐wide association studies
IVW: inverse variance weighting
MR: Mendelian randomization
NHANES: National Health and Nutrition Examination Survey
OR: odds ratios
PHQ‐9: patient health questionnaire‐9
PTSD: post‐traumatic stress disorder
RCS: restricted cubic spline
SD: standard deviation
SMI: severe mental illnesses
SNP: single nucleotide polymorphism
UACR: a ratio of albumin to creatinine

## 1 Background

Albuminuria is defined as urinary albumin excretion exceeding 30 mg/24 h or a ratio of albumin to creatinine (UACR) over 30 mg/g (Ruilope et al. 2023). Generally, UACR ranging from 30 to 300 mg/g is classified as microalbuminuria, whereas UACR exceeding 300 mg/g is categorized as macroalbuminuria (de Jong et al. 2007). Research indicates that patients face an elevated risk of developing chronic kidney disease (CKD) as urinary albumin concentrations increase (Roscioni et al. 2014; Bakris and Molitch 2014). Moreover, albuminuria is also considered a hallmark of microvascular damage (Boorsma et al. 2023). Evidence suggests a close association between albuminuria and various cardiovascular diseases such as atherosclerosis, hypertension, renal microvascular disease, and cerebral vascular lesions, which may be related to endothelial damage (Boorsma et al. 2023; Barzilay et al. 2024; Georgakis et al. 2018). Therefore, albuminuria is crucial in clinical medicine, serving as a key indicator for evaluating kidney and cardiovascular function.

Mental illness is complex, especially in the context of other illnesses. There is often a close association between cardiovascular or kidney diseases and mental illnesses. For instance, individuals with mental illnesses often experience various chronic conditions, such as kidney diseases and cardiovascular diseases, also accompanied by an increase in albuminuria (Cogley et al. 2022). An observational study suggested that schizophrenia may elevate the risk of CKD (Tzeng et al. 2015). Recent studies also indicated a higher prevalence of CKD among patients with severe mental illnesses (SMI), including conditions such as schizophrenia and bipolar disorder (Iwagami et al. 2018; Carswell et al. 2023). Moreover, patients with CKD are more prone to suffer from depression (Finkelstein et al. 2010). What's worse, patients with SMI who coexist with CKD frequently experience poorer outcomes and reduced quality of life (Carswell et al. 2023). Besides, a strong correlation also exists between mental illnesses and cardiovascular diseases (Tuttle 2004). Observational studies have found that patients with schizophrenia may have a higher risk of cardiovascular diseases, such as coronary artery disease (McCreadie and Scottish Schizophrenia Lifestyle Group 2003; Vance et al. 2019). Patients with depression have a higher rate of cardiovascular event mortality, and the two conditions often coexist (Pina et al. 2018; Schottke and Giabbiconi 2015). Further, patients with bipolar disorder often experience coronary microvascular dysfunction (Kennedy et al. 2023). It is worth noting that reports have shown that psychological factors such as depression and anxiety increase the risk of albuminuria 2–3 times (Gustad et al. 2022). Further, patients with albuminuria also demonstrate a tendency to have SMI (Carswell et al. 2023). These studies suggest that albuminuria seems to increase the risk of mental illness.

Unfortunately, these above associations are often bidirectional and complex, with relationships and underlying mechanisms still a puzzle. Identifying commonalities among them may be the key to preventing and solving mental illness. There are some observational evidences indicating a potential shared pathogenic mechanism between albuminuria and cognitive function in the brain (Huang et al. 2017) and a higher levels of albuminuria associated with a decline in cognitive function (McQuillan and Jassal 2010). Therefore, albuminuria may also serve as a potential risk factor for mental illnesses. However, there are no studies that link the two within the scope of current searches in the medical databases.

In this study, we conducted an observational study using US population data from the National Health and Nutrition Examination Survey (NHANES) 2005–2018, focusing on depression. Additionally, Mendelian randomization (MR) analysis was performed to uncover the effects of albuminuria on mental illness, including anxiety disorders, persistent delusional disorders, schizophrenia, schizotypal personality disorders, panic disorder, post‐traumatic stress disorder (PTSD), obsessive‐compulsive disorder, bipolar I disorder, bipolar II disorder, depression, autism, and social phobia. Briefly, our research aims to investigate the potential relationship between albuminuria and the risk of mental illness from population and genetic perspectives, as well as to explore the unique value of albuminuria as a clinical biomarker.

## 2 Methods

### 2.1 Study Population in NHANES

**FIGURE 1:**
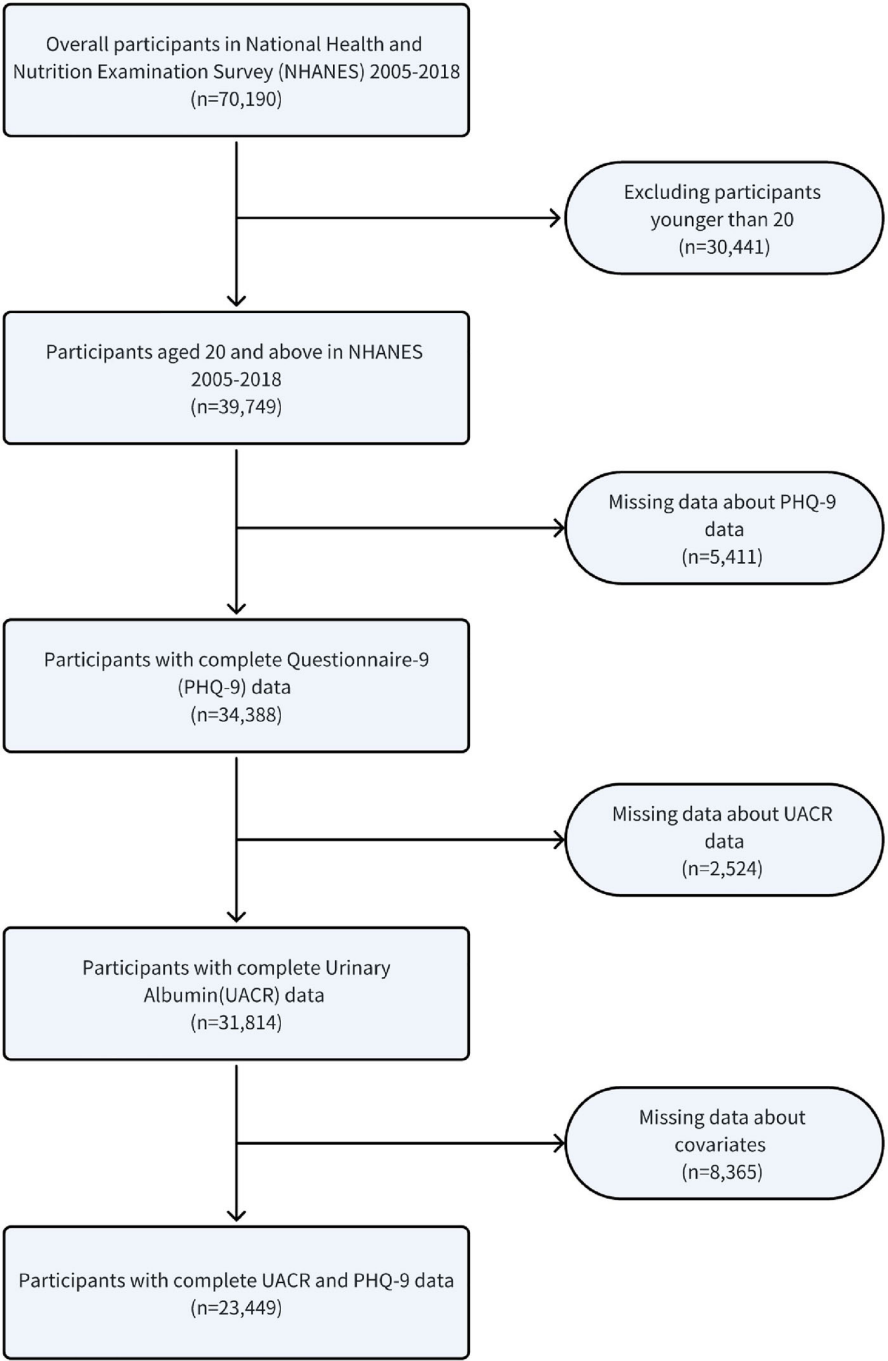
**Study design in NHANES**. Flowchart of sample selection from NHANES (2005–2018).

The data are derived from NHANES 2005–2018, which offers publicly available, nationally representative health and nutrition data for non‐institutionalized individuals in the United States. NHANES employs complex multistage and stratified probability sampling techniques. All participants provided informed consent for the surveys and examinations, and comprehensive information is accessible at https://www.cdc.gov/nchs/nhanes. Due to age restrictions on specific questionnaires, our study in the NHANES 2005–2018 dataset only included individuals aged 20 and above, thus focusing on US adults. Specifically, we initially enrolled 70,190 participants, with 38,376 individuals excluded from the study: those aged <20 years (*n* = 30,441), unable to complete the patient health questionnaire‐9 (PHQ‐9) (*n* = 5411), lacking measurements of urine albumin and creatinine concentrations (*n* = 2524), and missing data on other covariates (*n* = 8365). Ultimately, 23,449 participants were included in this study, and the screening process is depicted in Figure 1.

### 2.2 Assessment of Albuminuria in NHANES

The urinary albumin concentration of subjects was determined using a solid‐phase fluorescent immunoassay method for human urinary albumin measurement, as described by Chavers et al. (1984). Fluorescent immunoassay is a noncompetitive double‐antibody method used to quantify albumin levels in urine. The urinary creatinine levels of subjects were measured using the Jaffe rate reaction to form a red creatinine‐picrate complex, subsequently analyzed with the Beckman Synchron CX3 clinical analyzer (Cho et al. 2013; Qin et al. 2022). All urine samples were obtained at standardized mobile examination centers. Albuminuria was defined as a UACR of “> 30 mg/g.” Microalbuminuria is classified as a UACR ranging from 30 to 300 mg/g, whereas macroalbuminuria is defined as a UACR exceeding 300 mg/g. Albuminuria was considered an outcome variable. To achieve a normal distribution, a Log2‐transformation was employed when analyzing serum UACR as a continuous variable.

### 2.3 Assessment of Mental Illness (Depression) in NHANES

We utilized the PHQ‐9 to screen for depression among participants in the NHANES 2005–2018 cohort. This questionnaire boasts high sensitivity and specificity in detecting depression (Kroenke et al. 2001). The PHQ‐9 categorizes responses into four modules (“0” = Not at all; “1” = Several days; “2” = More than half the days; “3” = Nearly every day), with a score range of 0–27. Building upon prior research, individuals were categorized with depression into four levels: Scores of “0–4” were classified as “no depression,” “5–9” as “mild depression,” “10–14” as “moderate depression,” and “≥15” as “moderately severe to severe depression” (Ballou et al. 2019). Given the clinical cutoff of PHQ‐9, participants were considered to score 10 or higher as individuals with depression, using this as the outcome variable in weighted multiple regression (Kroenke et al. 2001).

### 2.4 Other Covariates in NHANES

The study also incorporated covariates potentially associated with depression, including age group (categorized as “20–39,” “40–59,” “60–79,” “80+”), gender, race (categorized as “Mexican American,” “Non‐Hispanic Black,” “Non‐Hispanic White,” “other Hispanic,” “other race—including multiracial”), education attainment (categorized as “Less Than 9th Grade,” “9–11th Grade (Includes 12th grade with no diploma),” “High School Grad/GED or Equivalent,” “Some College or Associate of Arts (AA) degree,” “College Graduate or above”), BMI (categorized as “Normal,” “Obese,” “Overweight,” “Underweight”), serum blood urea nitrogen (nmol/L), serum creatinine (mg/dL), alcohol consumption (“1–5 drinks/month,” “5–10 drinks/month,” “10+ drink/month,” “nondrinker”), smoking habits (“Current smoker,” “Former smoker,” “NO smoker”), and the presence of hypertension, coronary heart disease, and diabetes (Qin et al. 2022; Nielsen et al. 2021; Nunes 2023; Wootton et al. 2020; Maina et al. 2023).

### 2.5 Genome‐Wide Association Studies (GWASs) Sources

The primary genetic tool utilized for albuminuria was derived from a large‐scale whole‐genome association study by CKDgen (*n* = 348,954, including 51,861 cases and 297,093 controls), predominantly comprising individuals of European descent (Teumer et al. 2019). The primary genetic tools for schizophrenia, bipolar disorder type I, bipolar disorder type II, anxiety disorder, autism, and panic disorder were derived from various European populations’ GWASs (Jiang et al. 2021; Pedersen et al. 2023; Brasher et al. 2023). Data on schizotypal personality disorder, persistent delusional disorder, obsessive‐compulsive disorder, PTSD, depression, and social phobia were obtained from the FinnGen database. The FinnGen database is maintained by the Finnish government or research institutions and includes a wealth of health, demographic, and other relevant data. These databases offer abundant research materials that can be utilized for epidemiological and clinical studies, and other academic analyses (Kurki et al. 2023). In our MR analysis, we focused the outcomes and exposures entirely on individuals of European descent. More detailed information could be found in Table S1.

### 2.6 Selection of Genetic Instrumental Variables

**FIGURE 2:**
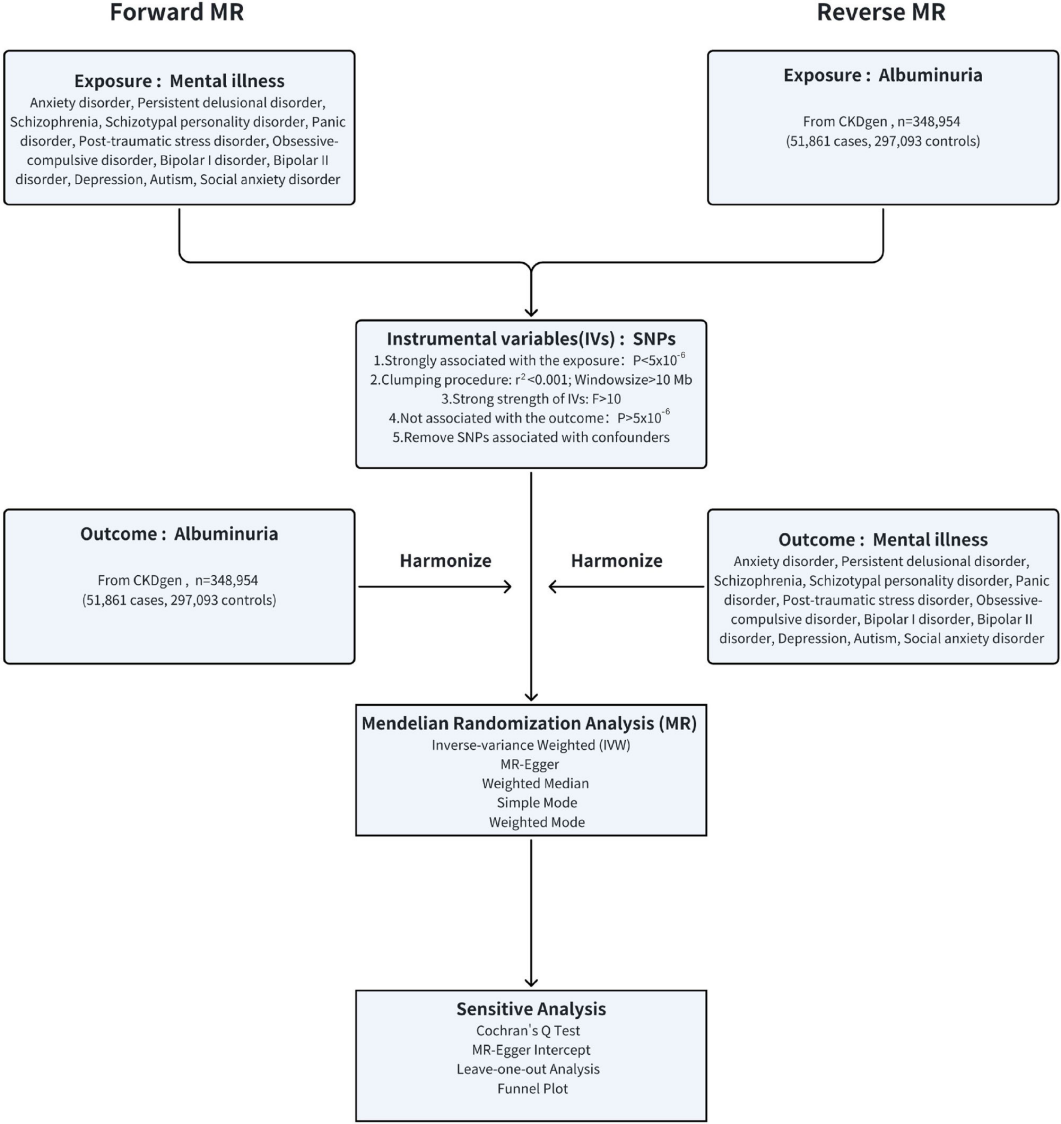
**Study design in MR**. Assumption **I**: IVs were strongly correlated with exposure. Assumption **II**: IVs were not associated with confounders. Assumption **III**: IVs were not associated with outcome.

Firstly, single nucleotide polymorphisms (SNPs) were screened highly associated with albuminuria (*p *< 5 × 10^−6^) and SNPs unrelated to the outcome (*p *< 5 × 10^−6^). Next, SNPs in linkage disequilibrium (LD) with *r* ^2^ < 0.001 and LD distance>10,000 kb were excluded. To acquire instrumental variables with sufficient statistical power, SNPs with an *F* statistic less than 10 were also removed. Lastly, taking into account the SNP's secondary phenotype, potential confounding SNPs that could influence the outcome were eliminated using Phenoscanner (www.phenoscanner.medschl.cam.ac.uk) (Figure 2).

### 2.7 Statistical Analysis

In the observational study of NHANES, the urine albumin concentration was stratified to describe the baseline characteristics of the overall population and corresponding strata participants. Categorical variables were presented as percentages, whereas continuous variables were expressed as means ± standard deviation (SD). Three weighted multivariate logistic regression models were employed to calculate the odds ratios (OR) and 95% confidence intervals (CI) for the risk of depression associated with albuminuria. Model 1 was unadjusted, whereas Model 2, built upon Model 1, adjusted for age, gender, race, and education attainment. Model 3, based on Model 2, additionally adjusted for BMI, serum blood urea nitrogen, serum creatinine, smoking, alcohol consumption, hypertension, coronary heart disease, and diabetes. Given the inclusion of seven cycles of NHANES data in our study, we recalculated the sample weights. Subgroup analyses were conducted to examine the relationship between albuminuria and depression, using stratification factors such as age group, gender, race, education attainment, diabetes, hypertension, and coronary heart disease. Additionally, interaction tests were employed to assess the consistency of this association across different subgroups. Restricted cubic spline (RCS) plots were used to explore the potential nonlinear relationship between these factors.

For the MR analysis, the inverse variance weighting (IVW) method was employed as the principal analytical approach (Yuan and Larsson 2022). In addition, MR‐Egger, weighted median, simple mode, and weighted mode methods were utilized to validate our results, all of which have been extensively discussed in previous studies (Li et al. 2023). Cochran's *Q* test was employed to assess the results of the IVW and MR‐Egger analyses (*p *< 0.05 indicating heterogeneity). The MR‐Egger Intercept test was utilized to examine horizontal pleiotropy (*p *< 0.05 suggesting potential pleiotropy) (Zhang et al. 2022). Furthermore, leave‐one‐out analysis and funnel plots were employed to validate the stability of our results. The “TwoSampleMR” package (version 0.5.7) in R (version 4.3.1) was utilized for MR analysis, with statistical significance defined as *p *< 0.05.

## 3 Results

### 3.1 Baseline Characteristics of the Participants

After a series of screenings, 23,449 participants were identified in the cross‐sectional study, whose weighted characteristics are depicted in Table 1 and Table S2. Following classification into four levels, participants were categorized as “no depression” (59.9%), “mild depression” (26.6%), “moderate depression” (8.2%), and “moderately severe to severe depression” (5.3%). On the basis of the clinical cutoff of PHQ‐9, the prevalence of depression in the included individuals was 13.5%.

**TABLE 1:** Baseline characteristics of the research population with and without depression.

	Non‐depression (*N* = 20,295)	Depression (*N* = 3154)	Total (*N* = 23,449)	*p* value
**Age group (%)**				**<0.001**
20–39	6830 (33.7%)	1022 (32.4%)	7852 (33.5%)	
40–59	6595 (32.5%)	1203 (38.1%)	7798 (33.3%)	
60–79	5670 (27.5%)	784 (24.9%)	6454 (27.5%)	
80+	1200 (5.9%)	145 (4.6%)	1345 (5.7%)	
**Age**				**0.038**
Mean (SD)	49.55 (17.73)	48.53 (16.64)	49.45 (17.59)	
**Sex (%)**				**< 0.001**
Female	9902 (48.7%)	1973 (62.6%)	11,875 (50.6%)	
Male	10,393 (51.2%)	1181 (37.4%)	11,574 (49.4%)	
**Race (%)**				**< 0.001**
Mexican American	3178 (15.7%)	495 (15.7%)	3673 (16.1%)	
Non‐Hispanic Black	4055 (20.0%)	647 (20.5%)	4702 (21.0%)	
Non‐Hispanic White	8790 (43.3%)	1355 (43.0%)	10,145 (43.9%)	
Other Hispanic	2129 (10.5%)	412 (13.1%)	2541 (9.6%)	
Other race—including multiracial	2143 (10.6%)	245 (7.8%)	2388 (9.3%)	
**Education attainment (%)**				**< 0.001**
Less than 9th grade	1969 (9.7%)	458 (14.5%)	2427 (10.4%)	
9–11th grade (includes 12th grade with no diploma)	2729 (13.4%)	651 (20.6%)	3380 (14.4%)	
High school grad/GED or equivalent	4555 (22.4%)	747 (23.7%)	5302 (22.6%)	
Some college or AA degree	5955 (29.3%)	935 (29.6%)	6890 (29.4%)	
College graduate or above	5087 (25.1%)	363 (11.5%)	5450 (23.2%)	
**BMI group (%)**				**< 0.001**
Normal	5596 (27.6%)	738 (23.4%)	6334 (27.0%)	
Obese	7439 (36.7%)	1509 (47.8%)	8948 (38.2%)	
Overweight	6975 (34.4%)	849 (26.9%)	7824 (33.4%)	
Underweight	285 (1.4%)	58 (1.8%)	343 (1.5%)	
**Serum creatinine (mg/dL)**				**< 0.001**
Mean (SD)	0.89 (0.35)	0.88 (0.47)	0.89 (0.37)	
**Blood urea nitrogen (mmol/L)**				
Mean (SD)	4.85 (2.05)	4.66 (2.33)	4.83 (2.09)	
**Smoking status (%)**				**< 0.001**
Current smoker	3736 (18.4%)	1113 (35.3%)	4849 (20.7%)	
Former smoker	4985 (24.6%)	707 (22.4%)	5692 (24.3%)	
NO smoker	11,574 (57.0%)	1334 (42.3%)	12,908 (55.0%)	
**Alcohol group (%)**				**< 0.001**
1–5 drinks/month	10,023 (49.4%)	1655 (52.5%)	11,678 (49.8%)	
5–10 drinks/month	1639 (8.1%)	213 (6.8%)	1582 (7.9%)	
10+ drink/month	2941 (14.5%)	407 (12.9%)	3348 (14.3%)	
Nondrinker	5692 (28.0%)	879 (27.9%)	6571 (28.0%)	
**Hypertension (%)**				**< 0.001**
False	13,276 (65.4%)	1726 (54.7%)	15,002 (64.0%)	
True	7019 (34.6%)	1428 (45.3%)	8447 (36.0%)	
**Coronary heart disease (%)**				**< 0.001**
False	19,552 (96.3%)	2984 (94.6%)	22,536 (96.1%)	
True	743 (3.7%)	170 (5.4%)	913 (3.9%)	
**Diabetes (%)**				**< 0.001**
False	17,844 (87.9%)	2645 (83.9%)	20,489 (87.4%)	
True	2451 (12.1%)	509 (16.1%)	2960 (12.6%)	
**UACR (mg/g)**				
Mean (SD)	41.40 (322.60)	69.00 (429.25)	45.11 (339.02)	**< 0.001**
**Albuminuria (%)**				**< 0.001**
False	17,992 (88.7%)	2646 (83.9%)	20,638 (88.0%)	
True	2303 (11.3%)	508 (16.1%)	2811 (12.0%)	
**Different types of albuminuria**				**< 0.001**
Non‐albuminuria	17,994 (88.7%)	2646 (83.9%)	20,640 (88.0%)	
Microalbuminuria	1900 (9.4%)	411 (13.0%)	2311 (9.9%)	
Macroalbuminuria	401 (2.0%)	97 (3.1%)	498 (2.1%)	

We found that individuals with depression were often aged 40–59, female, of other Hispanic descent, obese, and current smokers. Moreover, having hypertension, coronary artery disease, diabetes, or albuminuria may increase the risk of depression (Table 1 and Table S2).

### 3.2 Risk of Albuminuria and Mental Illness (Depression) in NHANES

The relationship between albuminuria and depression was investigated through three different multivariate logistic regression models. Regardless of model adjustments, a significant association was found between albuminuria and the risk of depression (Model 1: OR (95% CI) = 1.51 (1.30–1.74), *p *< 0.001; Model 2: OR (95% CI) = 1.46 (1.25–1.70), *p *< 0.001; Model 3: OR (95% CI) = 1.26 (1.08–1.47), *p* = 0.004).

Specifically, patients with microalbuminuria have a higher risk of depression (Model 1: OR (95% CI) = 1.49 (1.27–1.75), *p *< 0.001; Model 2: OR (95% CI) = 1.44 (1.22–1.70), *p *< 0.001; Model 3: OR (95% CI) = 1.27 (1.07–1.50), *p* = 0.006).

In the analysis of albuminuria as a continuous variable, a significant positive association between albuminuria and depression was also found (Model 1: OR (95% CI) = 1.15 (1.11–1.20), *p *< 0.001; Model 2: OR (95% CI) = 1.12 (1.08–1.17), *p *< 0.001; Model 3: OR (95% CI) = 1.06 (1.01–1.11), *p* = 0.023; Table 2).

**TABLE 2:** Associations among different types of albuminuria and the prevalence of depression.

	Model 1, OR (95% CI) *p*	Model 2, OR (95% CI) *p*	Model 3, OR (95% CI) *p*
**Log_2_‐transformed UACR (mg/g)**	1.15 (1.11–1.20) *p *< 0.001	1.12 (1.08–1.17) *p *< 0.001	1.06 (1.01–1.11) *p* = 0.023
**Albuminuria**			
Non‐albuminuria	Reference	Reference	Reference
Albuminuria	1.51 (1.30–1.74) *p *< 0.001	1.46 (1.25–1.70) *p *< 0.001	1.26 (1.08–1.47) *p* = 0.004
**Different types of albuminuria**			
Non‐albuminuria	Reference	Reference	Reference
Microalbuminuria	1.49 (1.27–1.75) *p *< 0.001	1.44 (1.22–1.70) *p *< 0.001	1.27 (1.07–1.50) *p* = 0.006
Macroalbuminuria	1.59 (1.20–2.10) *p* = 0.001	1.57 (1.18–2.1) *p* = 0.002	1.18 (0.87–1.61) *p* = 0.259

*Note*: Logistic regression models: Model 1: no covariates were adjusted. Model 2: was adjusted for age, gender, race, education attainment. Model 3: was adjusted for age, gender, race, education attainment, BMI, serum blood urea nitrogen, serum creatinine, smoking, alcohol consumption, hypertension, coronary heart disease, and diabetes.

Abbreviations: CI, confidence intervals; OR, odds ratios.

In the subgroup of non‐Hispanic Black females, 60–79 group, with the college or AA degree, there is a correlation between albuminuria and depression. Further, a strong correlation between albuminuria and depression was observed in individuals with hypertension, coronary heart disease, and diabetes. It is noteworthy that this trend becomes more pronounced with worsening symptoms of albuminuria. Interaction tests indicate that the relationship between albuminuria and depression is independent of the aforementioned factors (*p* > 0.05, Figures S1–S3).

**FIGURE 3:**
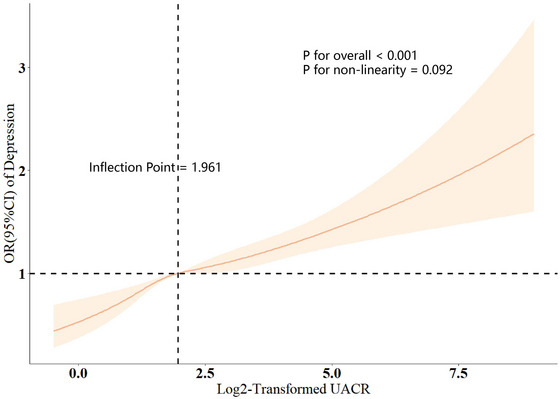
**RCS analysis of log2‐transformed UACR and odds ratio of depression based on Model 3**. Association between Log2‐Transformed UACR and depression. The odds ratio (OR) was estimated using the restricted cubic spline (RCS) analysis of Log2‐transformed UACR and odds ratio of depression based on Model 3. The horizontal bars represent 95% confidence intervals (CI).

In RCS analysis, the study found no evidence of a non‐linear relationship between the risk of depression and log2‐transformed serum UACR values (*p* for nonlinearity = 0.092, Figure 3). Instead, a linear dose–response relationship was observed between log2‐transformed serum UACR and depression risk.

### 3.3 MR Analysis of Albuminuria and Mental Illness

**FIGURE 4:**
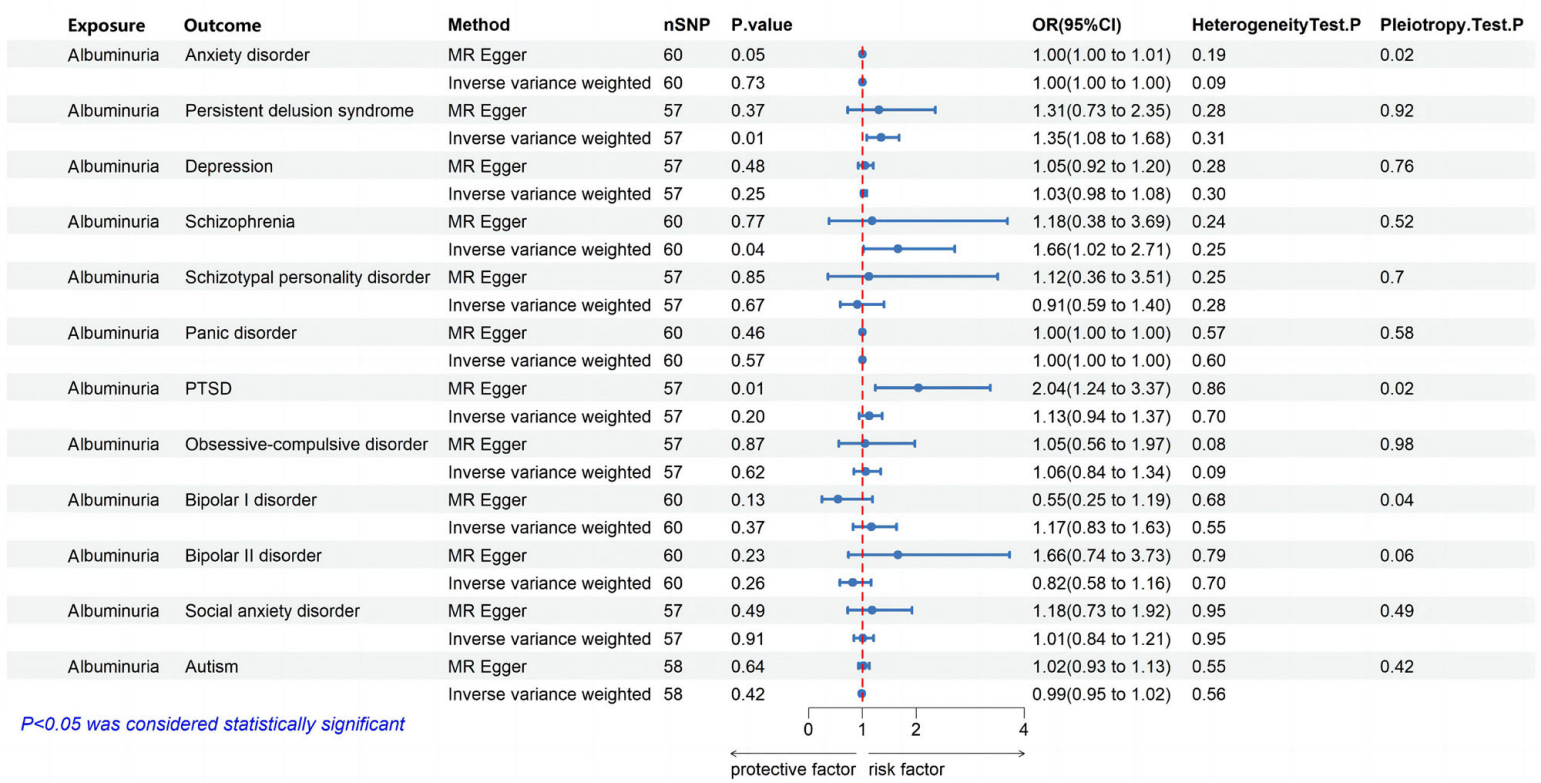
**Causal estimates of albuminuria on Mental illness by MR analysis**. Causal estimates of albuminuria on mental illness by MR analysis (IVW, MR‐Egger). Forest plots showing causal estimates of albuminuria on mental illness. Forest plots showing causal effects of albuminuria on mental illness. The odds ratio (OR) was estimated using the fixed effect IVW method. The horizontal bars represent 95% confidence intervals (CI). The heterogeneity tests represent the results of the IVW and MR‐Egger analyses by Cochran's *Q* test. The MR‐Egger intercept tests represent horizontal pleiotropy.

We identified a series of mental illness (anxiety disorder, persistent delusional disorder, schizophrenia, schizotypal personality disorder, panic disorder, PTSD, obsessive–compulsive disorder, bipolar I disorder, bipolar II disorder, depression, autism, and social phobia) as genetic instrumental variables for microalbuminuria, with specific SNP details listed in Table S3. IVW was the primary analytical method, with MR‐Egger, weighted median, simple mode, and weighted mode methods serving as supplementary results without impacting the IVW outcomes. We found no significant relationship between albuminuria and depression from genetic perspective (IVW: OR = 1.03, CI: 0.98–1.08, *p* = 0.25). Further, albuminuria increases the potential risk of developing persistent delusional disorder (IVW: OR = 1.35, CI: 1.08–1.67, *p *< 0.01; Figure 4 and Figure S4 and Table S4) and schizophrenia (IVW: OR = 1.66, CI: 1.02–2.71, *p* = 0.04; Figure 4 and Figure S4 and Table S4). Sensitivity analyses using Cochran's *Q* test and MR‐Egger Intercept test revealed no heterogeneity or pleiotropy in the results (*p* > 0.05, Table S5). Additionally, validation was further supported by leave‐one‐out analysis and funnel plots (Figures S5 and S6). In the reverse MR analysis, we did not find any significant relationship between the included mental illness and albuminuria, confirming the unidirectionality of the results (*p* > 0.05, Table S6).

## 4 Discussion

Albuminuria serves as a biomarker for predicting and assessing cardiorenal diseases. However, there is currently limited understanding of its role in mental disorders. Previous studies have often overlooked its significance, frequently treating it as a mere complication (Tsai et al. 2012). Our study aims to explore the potential of albuminuria as a biomarker in more depth. In our cross‐sectional study, albuminuria increases the risk of depression by 26%. Albuminuria as a continuous variable is also associated with depression. Moreover, subgroup analysis was used to enhance the understanding of these associations. Interestingly, we discovered a stronger correlation between albuminuria and depression in women. This finding may help explain the significantly higher prevalence of depression among women (62.6%) compared to men (37.4%). Although traditionally believed, the incidence of depression in women is often higher than in men (typically in a ratio of 2:1) (Neitzke 2016). The underlying causes of this disparity are typically multifaceted. In the study, albuminuria is regarded as a potential contributing factor of depression in this context.

Next, we identified albuminuria as a potential risk factor for schizophrenia in MR analysis. Previous observational studies have shown that patients with severe mental disorders often exhibit a higher prevalence of CKD and elevated urine albumin levels (Carswell et al. 2023). The relationship between schizophrenia and CKD is particularly close. Although not confirmed, patients with schizophrenia commonly exhibit an increased risk of CKD (Tzur Bitan et al. 2019). The fact to consider here is that mental illness is not only associated with decreased kidney function but also intricately linked to cardiovascular diseases. Previous observational research also has demonstrated that cardiovascular disease is the leading cause of mortality among individuals with severe mental illness, while revealing an association between severe mental illness and a higher incidence of cardiovascular disease (Vance et al. 2019). There is an evidence indicating that individuals with cardiovascular diseases have a higher risk of developing mental illnesses (Amarasekera and Jha 2022). However, these above perspectives are often ambiguous and bidirectional, overlooking potential underlying causal relationships. In our MR analysis, we revealed albuminuria as a potential risk factor for schizophrenia, providing a novel standpoint for exploring the connections among declining kidney function, cardiovascular diseases, and mental disorders.

Further, in the MR analysis, we also found albuminuria as a potential risk factor for persistent delusional disorder. It is worth noting that, on one side, the relationship between persistent delusional disorder and schizophrenia is intricately connected, creating a concept that is considered somewhat contentious in the field (Munoz‐Negro et al. 2015). The diagnosis of persistent delusional disorder lacks stability, often leading patients to transition into schizophrenia (Opjordsmoen 2014). In a cross‐sectional study, some scholars have characterized it as mild symptoms of schizophrenia, indicating a subtle form of the illness (Munoz‐Negro et al. 2018). On another side, in clinical settings, schizophrenia often co‐occurs with depression, leading to patients exhibiting not only overlapping symptoms but also inseparable domains of symptoms (Herniman et al. 2019). For our study, albuminuria leaded to an increased risk of both schizophrenia and persistent delusional disorder at the genetic level, suggesting potential shared pathogenic mechanisms between them.

Here we have linked albuminuria with mental illness and examined the relationship between them. These findings have the following implications: (1) Mental illnesses are significantly influenced by lifestyle habits and environmental factors (Goldfarb et al. 2022). Albuminuria could be served as an independent risk factor for the early intervention in promoting the mental well‐being of albuminuria patients. (2) It may present a novel method for screening schizophrenia and persistent delusional disorder, thereby reducing subjective confusion among patients. (3) Previous evidence indicates an association between second‐generation antipsychotic medications and the progression of kidney disease (Hojlund et al. 2020; Wang et al. 2018). For individuals with schizophrenia or persistent delusional disorder accompanied by significant renal impairment, personalized treatment should consider reinforcing kidney protective measures. Doctors should opt for medications that are less taxing on kidney function or adjust dosages to alleviate adverse effects on the kidneys. When treating schizophrenia or persistent delusional disorder, simultaneous attention to and management of renal function can provide a comprehensive understanding of the patients’ disease status, enhance treatment efficacy, and reduce the incidence of adverse events.

Our study has several strengths. Firstly, NHANES exhibits high reliability and generalizability, facilitating the derivation of more trustworthy results. We used a multivariable weighted logistic regression model for our study, while also examining the issue of multicollinearity among covariates. To ensure the stability and objectivity of our findings, we combined cross‐sectional analysis with MR analysis. MR analysis, an epidemiological analytical method, also known as nature's randomized controlled study, utilizes genetic variations (SNPs) as instrumental variables to evaluate the relationship between exposure factors and outcome events, providing more trustworthy research conclusions (Jung et al. 2020; Ponsford et al. 2020). Moreover, we employed multivariable logistic regression, subgroup analysis, interaction tests, sensitivity analysis, and heterogeneity tests to further enhance the credibility and accuracy of our results. However, our study also has limitations. First, in the cross‐sectional study, much data were obtained through questionnaires, which may be susceptible to various biases. Secondly, our MR analysis focused primarily on a European population, limiting the generalizability of the results to other populations. Although we stratified the study population by different subgroups in the cross‐sectional analysis, the lack of specific data in the GWAS dataset required us to further confirm our experimental conclusions. Schizophrenia is an early onset neurological disorder, whereas albuminuria is commonly found in middle‐aged and elderly patients (Jauhar et al. 2022; Ingelsson et al. 2007). It is crucial to consider the temporal sequence of the occurrence of these conditions. In our study, we recognized the limitations of traditional observational research due to potential time‐related confounders and, therefore, incorporated reverse MR analysis (Smith and Ebrahim 2003). Specifically, we utilized MR analysis to investigate the relationship between albuminuria and mental disorders, including schizophrenia, using both forward and reverse analyses. This approach offers new insights into explaining the relationship between albuminuria and mental disorders. Although this method employs genetic variations as instrumental variables to identify causal relationships and reduce biases commonly seen in observational designs, it does not entirely address confounding factors and time effects (Holmes and Davey Smith 2019). Unfortunately, the lack of detailed databases hinders in‐depth analysis to dissect the specific role of albuminuria. Nonetheless, we remain committed to exploring the unique biological effects of albuminuria and aim to elucidate these intricate relationships in future studies.

## 5 Conclusion

Our results confirmed that albuminuria is positively associated with the risk of depression. We also observed a potential relationship between albuminuria and schizophrenia, as well as persistent delusional disorder.

## Author Contributions

**Yangyang Wang**: writing–original draft, writing–review and editing, visualization, methodology, conceptualization, software, data curation, formal analysis, validation, investigation. **Sen Li**: funding acquisition, writing–review and editing, supervision, resources, project administration, investigation, validation.

## Ethics Statement

The studies involving human participants were granted ethical approval by the NCHS Research Ethics Review Board. These studies were carried out in compliance with local legislation and institutional requirements. Prior to participation in this study, all participants provided written informed consent. Our research was conducted using publicly available anonymized databases, namely, GWAS and FinnGen, which are exempt from ethical compliance.

## Consent

The authors have nothing to report.

## Conflicts of Interest

The authors declare no conflicts of interest.

### Peer Review

The peer review history for this article is available at https://publons.com/publon/10.1002/brb3.70545

## Supporting information




**Figure S1 Subgroup analysis for the association between albuminuria and depression**. Association between albuminuria and depression by full‐adjusted weighted multivariate logistic regression models. (**A)** Forest plots showing association between albuminuria and depression. (**B)** Association between albuminuria and depression. The odds ratio (OR) was estimated using the full‐adjusted weighted multivariate logistic regression models. The horizontal bars represent 95% confidence intervals (CI). (**C)** The P for interaction represents the results of the interaction tests.

**Figure S2 Subgroup analysis for the association among different types of albuminuria and depression**. Association among different types of albuminuria and depression by full‐adjusted weighted multivariate logistic regression models. Association among different types of albuminuria and depression by full‐adjusted weighted multivariate logistic regression models. (**A)** Forest plots showing association among different types of albuminuria and depression. (**B)** Association among different types of albuminuria and depression. The odds ratio (OR) was estimated using the full‐adjusted weighted multivariate logistic regression models. The horizontal bars represent 95% confidence intervals (CI). (**C)** The P for interaction represents the results of the interaction tests.

**Figure S3 Subgroup analysis for the association between UACR and depression** association between Log2‐transformed UACR (mg/g) and depression by full‐adjusted weighted multivariate logistic regression models. Association between Log2‐Transformed UACR and depression by full‐adjusted weighted multivariate logistic regression models. (**A)** Forest plots showing association between Log2‐transformed UACR and depression. (**B)** Association between Log2‐transformed UACR and depression. The odds ratio (OR) was estimated using the full‐adjusted weighted multivariate logistic regression models. The horizontal bars represent 95% confidence intervals (CI). (**C)** The P for interaction represents the results of the interaction tests.

**Figure S4 Scatter plots for MR analyses of the causal effect of albuminuria on mental illness**. The MR analyses were carried out utilizing various methods, including fixed‐effect inverse variance weighting, weighted mean, MR‐Egger, weight mode, and weight median. Each line's slope represents the estimated MR effect for the specific method, with error bars indicating the 95% confidence intervals around each SNP: (A) anxiety disorder, (B) persistent delusional disorder, (C) depression, (D) schizophrenia, (E) schizotypal personality disorder, (F) panic disorder, (G) post‐traumatic stress disorder, (H) obsessive‐compulsive disorder, (I) bipolar I disorder, (J) bipolar II disorder, (K) social anxiety disorder, and (L) autism.

**Figure S5 Funnel plots for the causal effect of albuminuria on mental illness**. Funnel plots were generated for Mendelian randomization (MR) analyses investigating the relationship between albuminuria and mental illness. These plots display the inverse variance weighted MR estimate for each albuminuria single‐nucleotide polymorphism with cytokines plotted against 1/standard error (1/SEIV): (A) anxiety disorder, (B) persistent delusional disorder, (C) depression, (D) schizophrenia, (E) schizotypal personality disorder, (F) panic disorder, (G) post‐traumatic stress disorder, (H) obsessive‐compulsive disorder, (I) bipolar I disorder, (J) bipolar II disorder, (K) social anxiety disorder, and (L) autism.

**Figure S6 Plots of leave‐one‐out analyses for the causal effect of albuminuria on mental illness**. Forest plots illustrating the causal estimates of albuminuria on mental illness by sequentially excluding each instrumental variable. The horizontal bars depict the beta value and its corresponding 95% confidence intervals (CI) for each causal estimate: (A) anxiety disorder, (B) persistent delusional disorder, (C) depression, (D) schizophrenia, (E) schizotypal personality disorder, (F) panic disorder, (G) post‐traumatic stress disorder, (H) obsessive‐compulsive disorder, (I) bipolar I disorder, (J) bipolar II disorder, (K) social anxiety disorder, and (L) autism.

**Table S1** Baseline characteristics of the research population with different types of depression.

**Table S2** The source and definition of exposure and outcome.

**Table S3** Inclusion of instrumental variables.

**Table S4** MR analysis.

**Table S5** Sensitivity analysis.

**Table S6** Reverse MR analysis.

Supporting Information.

Supporting Information.

## Data Availability

All data generated or analyzed during this study are included in this published article and its Supporting Information files.
