# Supplementary figures and images for: Albuminuria and Mental Illness Risk: Results From National Health and Nutrition Examination Survey 2005–2018 and Mendelian Randomization Analyses
Source: Brain Behav. 2025 May 11;15(5):e70545. doi: 10.1002/brb3.70545 (PMC12066806; doi:10.1002/brb3.70545)

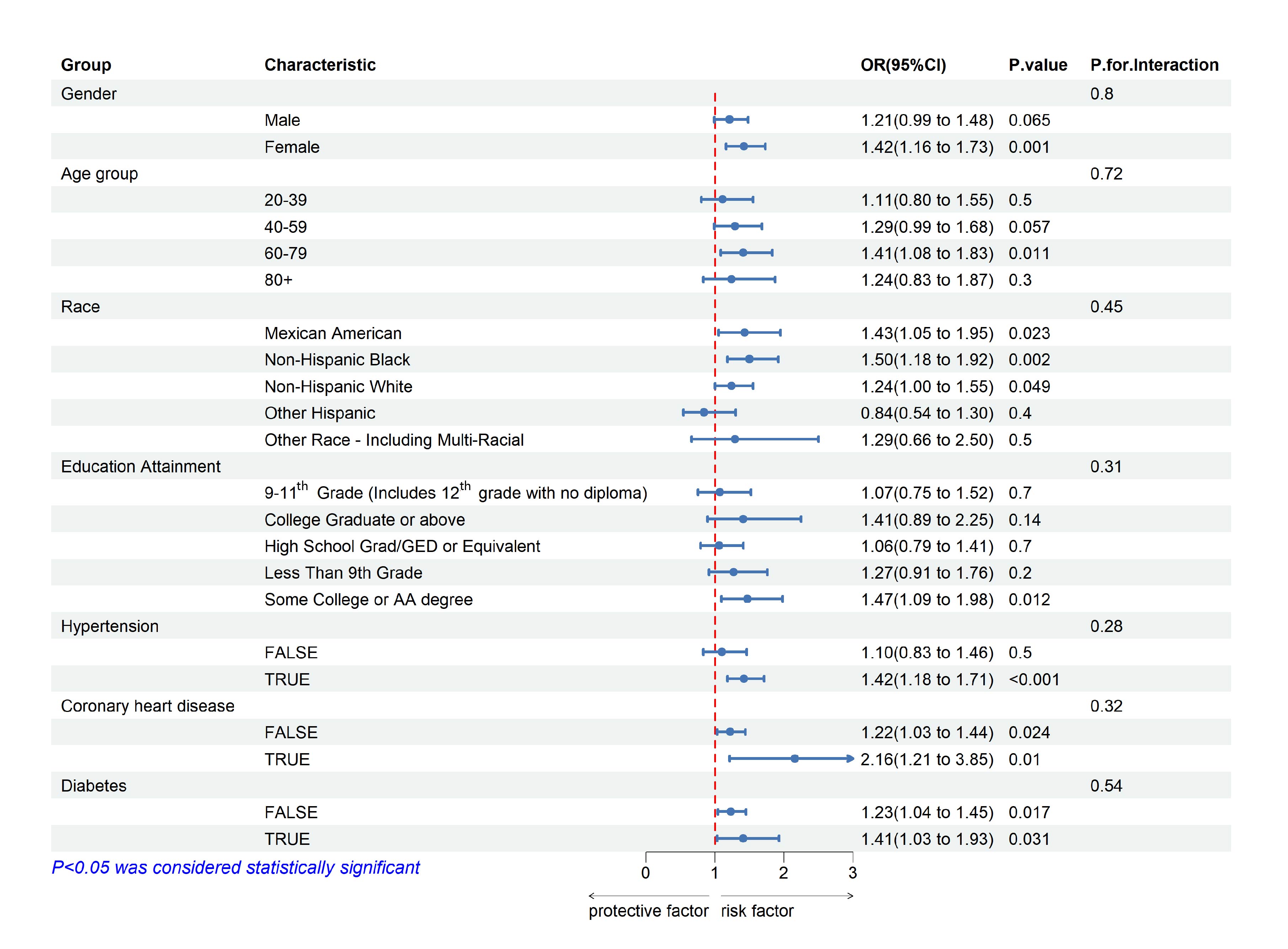

Supplement: Supplementary file 1 — Figure S1 Subgroup analysis for the association between albuminuria and depression. Association between albuminuria and depression by full‐adjusted weighted multivariate logistic regression models. (A) Forest plots showing association between albuminuria and depression. (B) Association between albuminuria and depression. The odds ratio (OR) was estimated using the full‐adjusted weighted multivariate logistic regression models. The horizontal bars represent 95% confidence intervals (CI). (C) The P for interaction represents the results of the interaction tests. [file BRB3-15-e70545-s009.jpg]

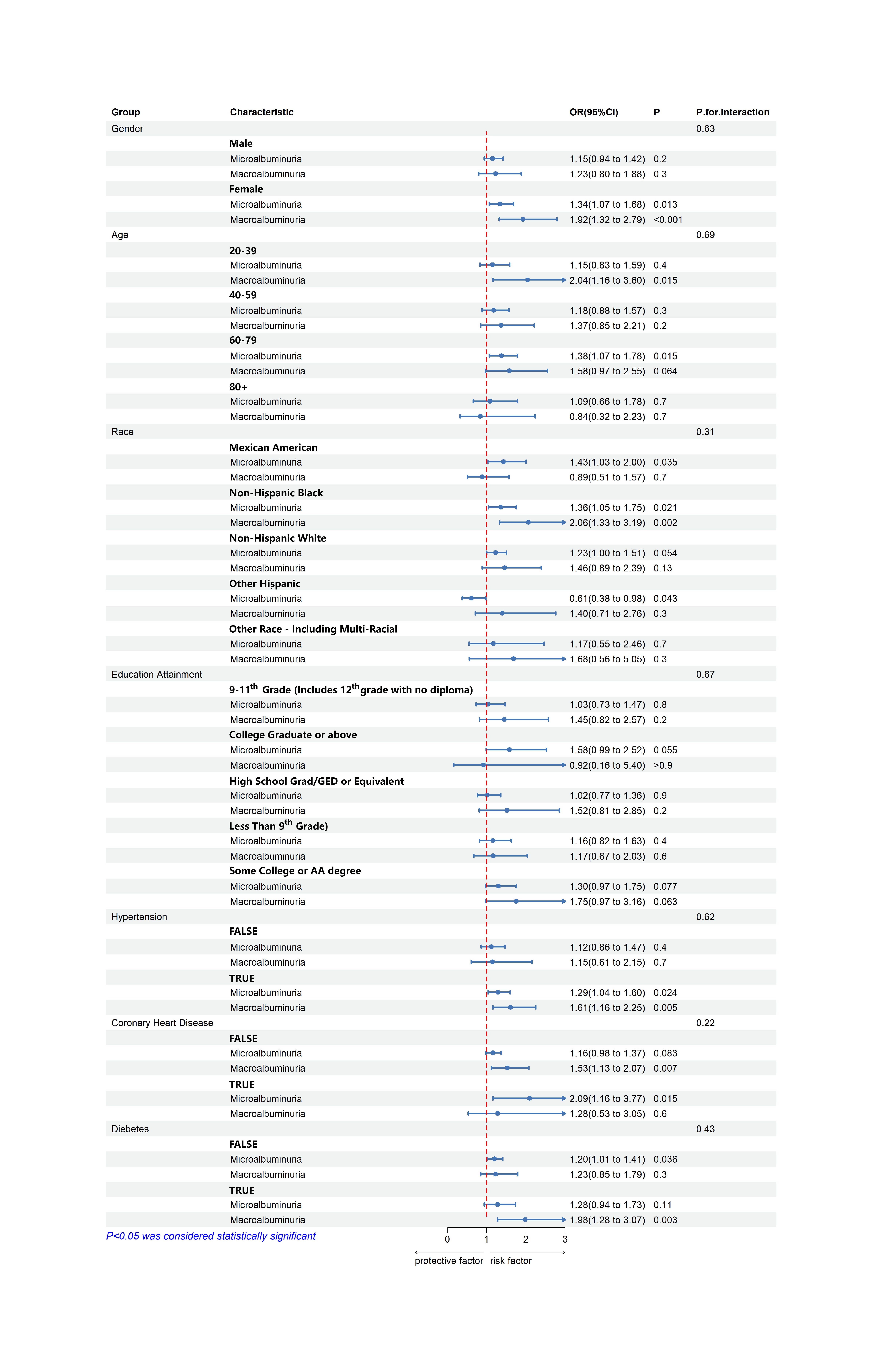

Supplement: Supplementary file 2 — Figure S2 Subgroup analysis for the association among different types of albuminuria and depression. Association among different types of albuminuria and depression by full‐adjusted weighted multivariate logistic regression models. Association among different types of albuminuria and depression by full‐adjusted weighted multivariate logistic regression models. (A) Forest plots showing association among different types of albuminuria and depression. (B) Association among different types of albuminuria and depression. The odds ratio (OR) was estimated using the full‐adjusted weighted multivariate logistic regression models. The horizontal bars represent 95% confidence intervals (CI). (C) The P for interaction represents the results of the interaction tests. [file BRB3-15-e70545-s011.jpg]

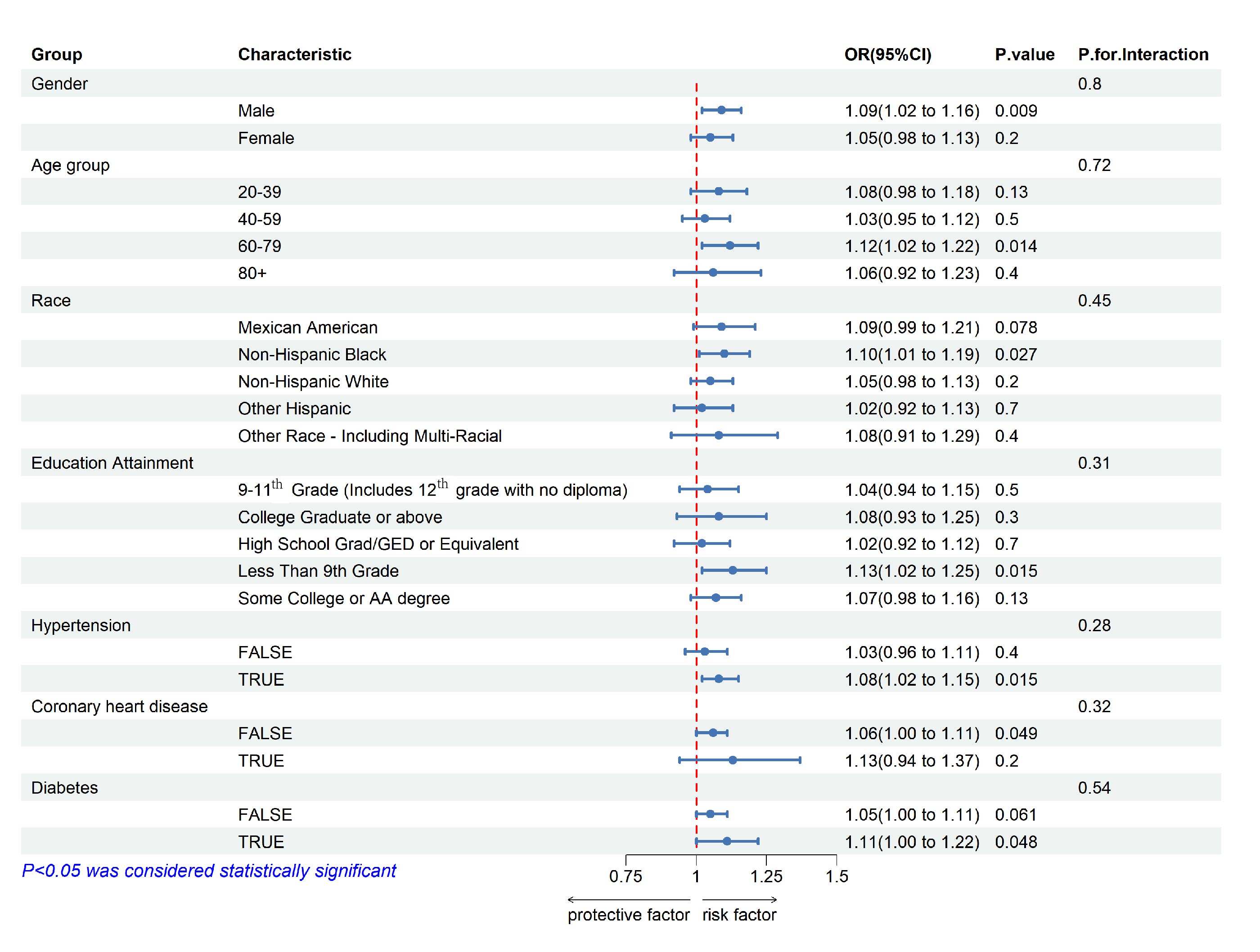

Supplement: Supplementary file 3 — Figure S3 Subgroup analysis for the association between UACR and depression association between Log2‐transformed UACR (mg/g) and depression by full‐adjusted weighted multivariate logistic regression models. Association between Log2‐Transformed UACR and depression by full‐adjusted weighted multivariate logistic regression models. (A) Forest plots showing association between Log2‐transformed UACR and depression. (B) Association between Log2‐transformed UACR and depression. The odds ratio (OR) was estimated using the full‐adjusted weighted multivariate logistic regression models. The horizontal bars represent 95% confidence intervals (CI). (C) The P for interaction represents the results of the interaction tests. [file BRB3-15-e70545-s013.jpg]

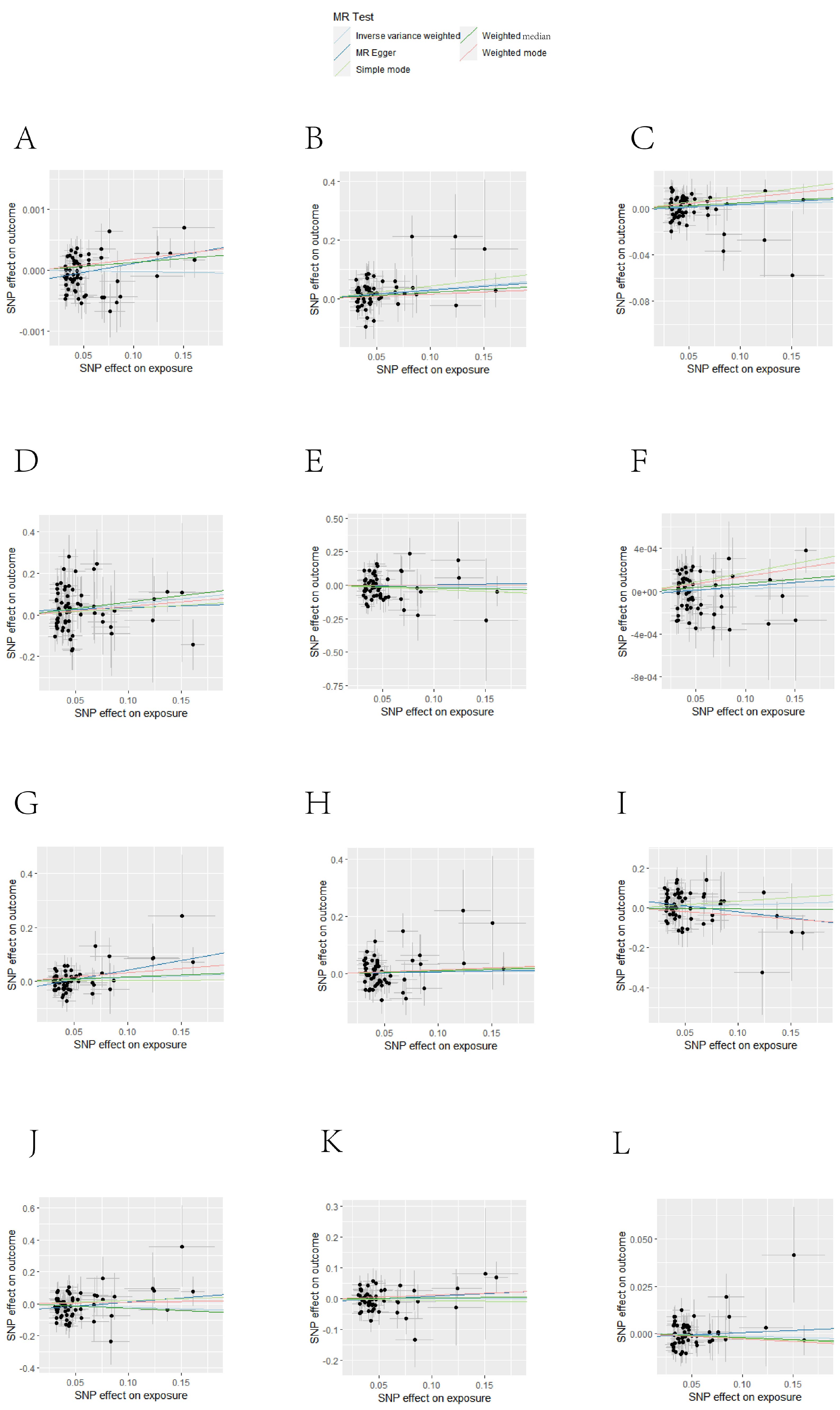

Supplement: Supplementary file 4 — Figure S4 Scatter plots for MR analyses of the causal effect of albuminuria on mental illness. The MR analyses were carried out utilizing various methods, including fixed‐effect inverse variance weighting, weighted mean, MR‐Egger, weight mode, and weight median. Each line's slope represents the estimated MR effect for the specific method, with error bars indicating the 95% confidence intervals around each SNP: (A) anxiety disorder, (B) persistent delusional disorder, (C) depression, (D) schizophrenia, (E) schizotypal personality disorder, (F) panic disorder, (G) post‐traumatic stress disorder, (H) obsessive‐compulsive disorder, (I) bipolar I disorder, (J) bipolar II disorder, (K) social anxiety disorder, and (L) autism. [file BRB3-15-e70545-s012.jpg]

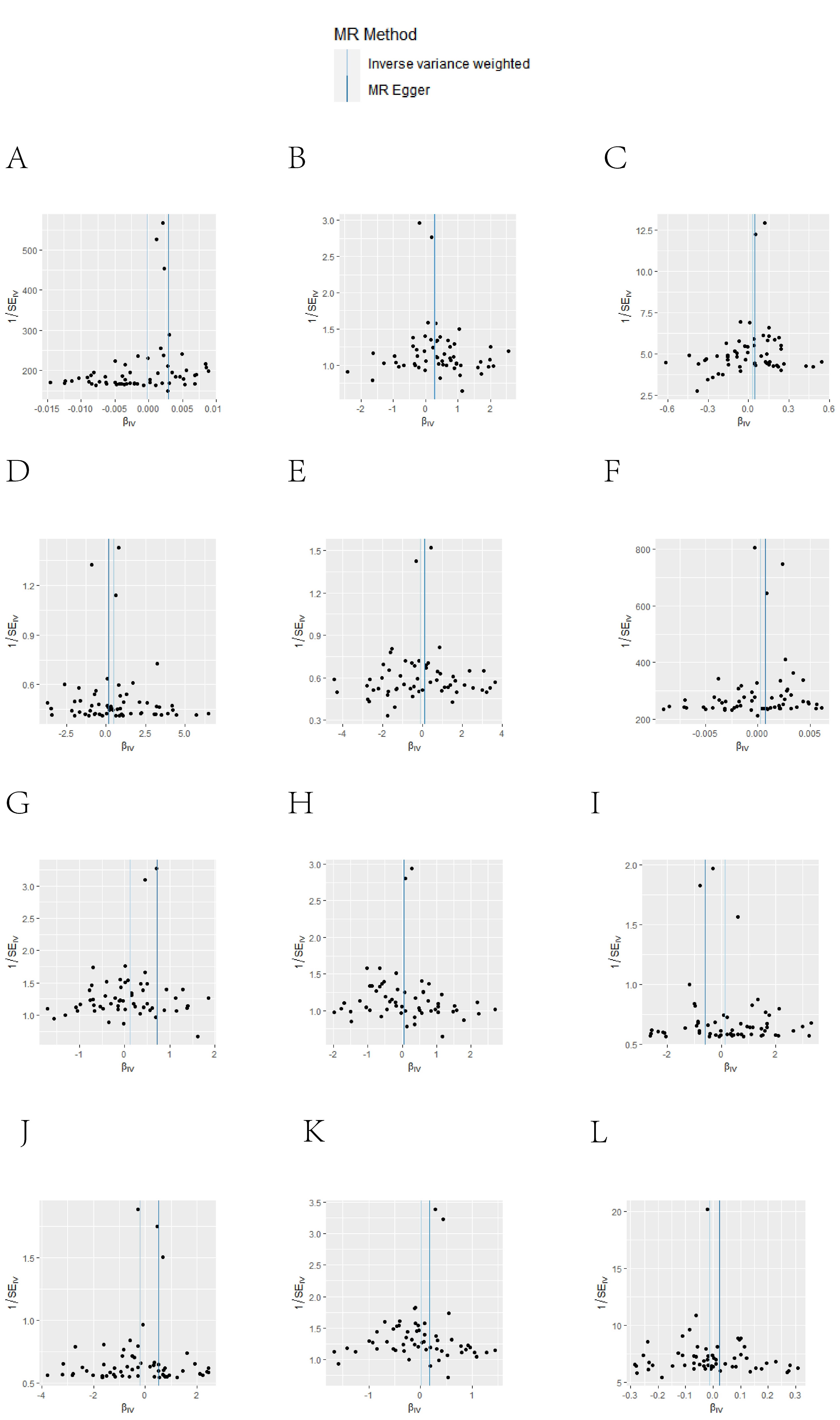

Supplement: Supplementary file 5 — Figure S5 Funnel plots for the causal effect of albuminuria on mental illness. Funnel plots were generated for Mendelian randomization (MR) analyses investigating the relationship between albuminuria and mental illness. These plots display the inverse variance weighted MR estimate for each albuminuria single‐nucleotide polymorphism with cytokines plotted against 1/standard error (1/SEIV): (A) anxiety disorder, (B) persistent delusional disorder, (C) depression, (D) schizophrenia, (E) schizotypal personality disorder, (F) panic disorder, (G) post‐traumatic stress disorder, (H) obsessive‐compulsive disorder, (I) bipolar I disorder, (J) bipolar II disorder, (K) social anxiety disorder, and (L) autism. [file BRB3-15-e70545-s001.jpg]

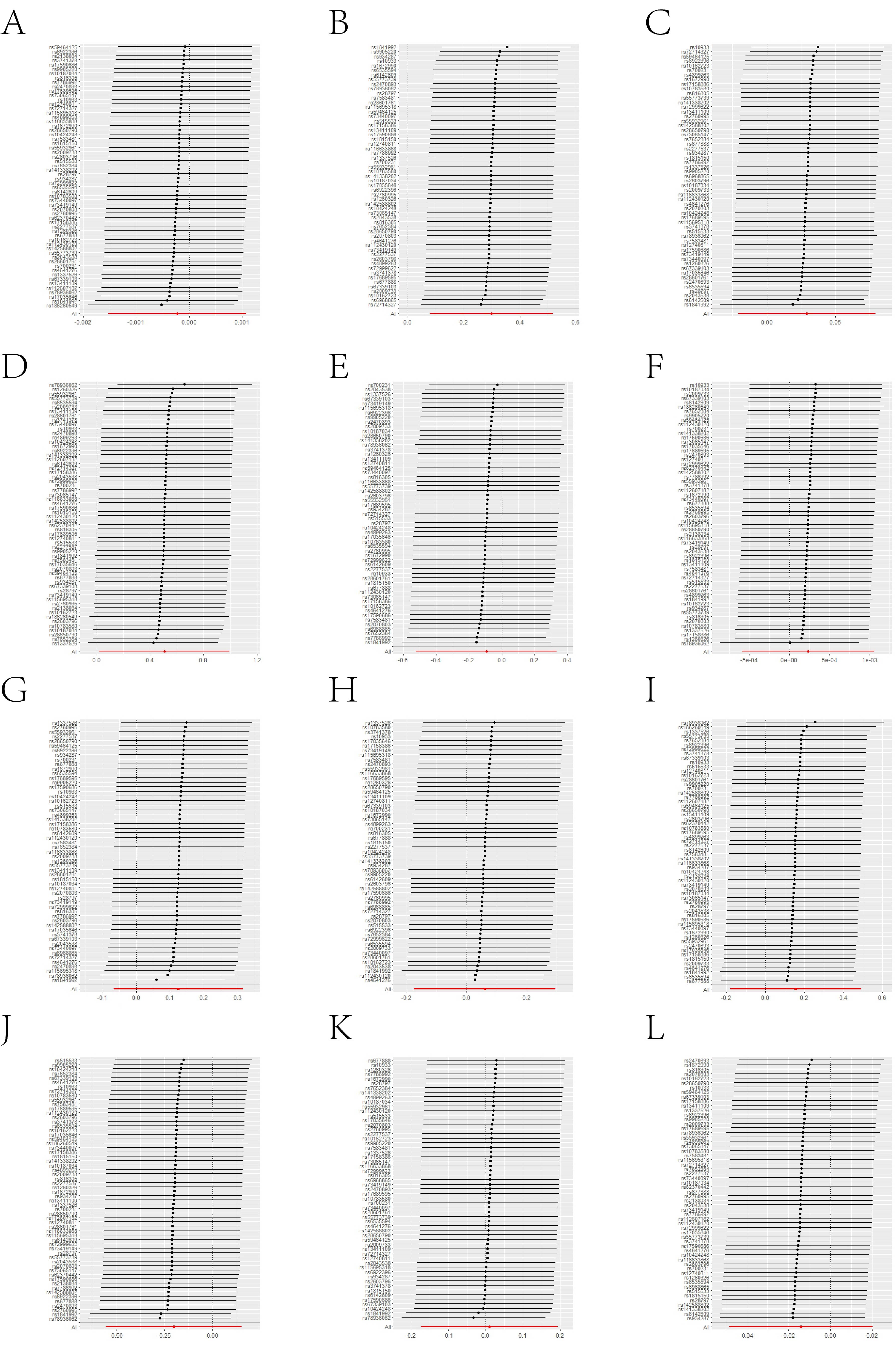

Supplement: Supplementary file 6 — Figure S6 Plots of leave‐one‐out analyses for the causal effect of albuminuria on mental illness. Forest plots illustrating the causal estimates of albuminuria on mental illness by sequentially excluding each instrumental variable. The horizontal bars depict the beta value and its corresponding 95% confidence intervals (CI) for each causal estimate: (A) anxiety disorder, (B) persistent delusional disorder, (C) depression, (D) schizophrenia, (E) schizotypal personality disorder, (F) panic disorder, (G) post‐traumatic stress disorder, (H) obsessive‐compulsive disorder, (I) bipolar I disorder, (J) bipolar II disorder, (K) social anxiety disorder, and (L) autism. [file BRB3-15-e70545-s002.jpg]
