# Supplementary material for: Albuminuria and Mental Illness Risk: Results From National Health and Nutrition Examination Survey 2005–2018 and Mendelian Randomization Analyses
Source: Brain Behav. 2025 May 11;15(5):e70545. doi: 10.1002/brb3.70545 (PMC12066806; doi:10.1002/brb3.70545)
Supplement: Supplementary file 8 — Table S2 The source and definition of exposure and outcome. [file BRB3-15-e70545-s007.docx]

## Table S2. Baseline Characteristics of the Research Population with Different Types of Depression

|  | **Mild Depression (N=6243)** | **Moderate Depression (N=1904)** | **Moderately Severe to Severe Depression (N=1250)** | **Non-Depression (N=14052)** | **Total (N=23449)** | **P.value** |
| --- | --- | --- | --- | --- | --- | --- |
| **Age group (%)** |  |  |  |  |  | <0.001 |
| 20-39 | 2251 (36.1%) | 663 (34.8%) | 359 (28.7%) | 4579 (32.6%) | 7852 (33.5%) |  |
| 40-59 | 2029 (32.5%) | 677 (35.6%) | 526 (42.1%) | 4566 (32.5%) | 7798 (33.3%) |  |
| 60-79 | 1594 (25.5%) | 466 (24.5%) | 318 (25.4%) | 4076 (29.0%) | 6454 (27.5%) |  |
| 80+ | 369 (5.9%) | 98 (5.1%) | 47 (3.8%) | 831 (5.9%) | 1345 (5.7%) |  |
| **Age** |  |  |  |  |  | <0.001 |
| Mean (SD) | 48.40 (17.94) | 48.38 (17.26) | 49.55 (15.63) | 50.06 (17.61) | 49.45 (17.59) |  |
| **Sex (%)** |  |  |  |  |  | <0.001 |
| female | 3475 (55.7%) | 1175 (61.7%) | 798 (63.8%) | 6427 (45.7%) | 11875 (50.6%) |  |
| male | 2768 (44.3%) | 729 (38.3%) | 452 (36.2%) | 7625 (54.3%) | 11574 (49.4%) |  |
| **Race (%)** |  |  |  |  |  | <0.001 |
| Mexican American | 973 (15.6%) | 309 (16.2%) | 186 (14.9%) | 2205 (15.7%) | 3673 (15.7%) |  |
| Non-Hispanic Black | 1227 (19.7%) | 392 (20.6%) | 255 (20.4%) | 2828 (20.1%) | 4702 (20.1%) |  |
| Non-Hispanic White | 2763 (44.3%) | 812 (42.6%) | 543 (43.4%) | 6027 (42.9%) | 10145 (43.3%) |  |
| other Hispanic | 677 (10.8%) | 227 (11.9%) | 185 (14.8%) | 1452 (10.3%) | 2541 (10.8%) |  |
| Other Race -Including Multi-Racial | 603 (9.7%) | 164 (8.6%) | 81 (6.5%) | 1540 (11.0%) | 2388 (10.2%) |  |
| **Education Attainment (%)** |  |  |  |  |  | <0.001 |
| Less Than 9^th^ Grade | 598 (9.6%) | 248 (13.0%) | 210 (16.8%) | 1371 (9.8%) | 2427 (10.4%) |  |
| 9-11^th^ Grade (Includes 12^th^ grade with no diploma) | 971 (15.6%) | 380 (20.0%) | 271 (21.7%) | 1758 (12.5%) | 3380 (14.4%) |  |
| High School Grad/GED or Equivalent | 1453 (23.3%) | 445 (23.4%) | 302 (24.2%) | 3102 (22.1%) | 5302 (22.6%) |  |
| Some College or AA degree | 1934 (31.0%) | 586 (30.8%) | 349 (27.9%) | 4021 (28.6%) | 6890 (29.4%) |  |
| College Graduate or above | 1287 (20.6%) | 245 (12.9%) | 118 (9.4%) | 3800 (27.0%) | 5450 (23.2%) |  |
| **BMI Group (%)** |  |  |  |  |  | <0.001 |
| Normal | 1611 (25.8%) | 483 (25.4%) | 255 (20.4%) | 3985 (28.4%) | 6334 (27.0%) |  |
| Obese | 2560 (41.0%) | 883 (46.4%) | 626 (50.1%) | 4879 (34.7%) | 8948 (38.2%) |  |
| Overweight | 1959 (31.4%) | 501 (26.3%) | 348 (27.8%) | 5016 (35.7%) | 7824 (33.4%) |  |
| Underweight | 113 (1.8%) | 37 (1.9%) | 21 (1.7%) | 172 (1.2%) | 343 (1.5%) |  |
| **Serum Creatinine (mg/dL)** |  |  |  |  |  | <0.001 |
| Mean (SD) | 0.88 (0.38) | 0.88 (0.44) | 0.89 (0.50) | 0.90 (0.33) | 0.89 (0.37) |  |
| **Blood Urea Nitrogen (mmol/L)** |  |  |  |  |  |  |
| Mean (SD) | 4.77 (2.20) | 4.68 (2.38) | 4.63 (2.26) | 4.89 (1.98) | 4.83 (2.09) |  |
| **Smoking Status (%)** |  |  |  |  |  | <0.001 |
| Current smoker | 1406 (22.5%) | 633 (33.2%) | 480 (38.4%) | 2330 (16.6%) | 4849 (20.7%) |  |
| Former smoker | 1480 (23.7%) | 419 (22.0%) | 288 (23.0%) | 3505 (24.9%) | 5692 (24.3%) |  |
| NO smoker | 3357 (53.8%) | 852 (44.7%) | 482 (38.6%) | 8217 (58.5%) | 12908 (55.0%) |  |
| **Alcohol Group (%)** |  |  |  |  |  | <0.001 |
| 1-5 drinks/month | 3155 (50.5%) | 985 (51.7%) | 670 (53.6%) | 6868 (48.9%) | 11678 (49.8%) |  |
| 10+ drink/month | 486 (7.8%) | 145 (7.6%) | 68 (5.4%) | 1153 (8.2%) | 1852 (7.9%) |  |
| 5-10 drinks/month | 895 (14.3%) | 258 (13.6%) | 149 (11.9%) | 2046 (14.6%) | 3348 (14.3%) |  |
| Non-drinker | 1707 (27.3%) | 516 (27.1%) | 363 (29.0%) | 3985 (28.4%) | 6571 (28.0%) |  |
| **Hypertension (%**) |  |  |  |  |  | <0.001 |
| FALSE | 3922 (62.8%) | 1074 (56.4%) | 652 (52.2%) | 9354 (66.6%) | 15002 (64.0%) |  |
| TRUE | 2321 (37.2%) | 830 (43.6%) | 598 (47.8%) | 4698 (33.4%) | 8447 (36.0%) |  |
| **Coronary Heart Disease (%)** |  |  |  |  |  | <0.001 |
| FALSE | 5991 (96.0%) | 1805 (94.8%) | 1179 (94.3%) | 13561 (96.5%) | 22536 (96.1%) |  |
| TRUE | 252 (4.0%) | 99 (5.2%) | 71 (5.7%) | 491 (3.5%) | 913 (3.9%) |  |
| **Diabetes (%)** |  |  |  |  |  | <0.001 |
| FALSE | 5528 (88.5%) | 1615 (84.8%) | 1030 (82.4%) | 12316 (87.6%) | 20489 (87.4%) |  |
| TRUE | 715 (11.5%) | 289 (15.2%) | 220 (17.6%) | 1736 (12.4%) | 2960 (12.6%) |  |
| **UACR (mg/g)** |  |  |  |  |  | <0.001 |
| Mean (SD) | 45.099 (336.43) | 67.640 (382.62) | 71.083 (492.02) | 39.767 (316.26) | 45.119 (339.02) |  |
| **Albuminuria (%)** |  |  |  |  |  | <0.001 |
| FALSE | 5508 (88.2%) | 1608 (84.5%) | 1038 (83.0%) | 12484 (88.8%) | 20638 (88.0%) |  |
| TRUE | 735 (11.8%) | 296 (15.5%) | 212 (17.0%) | 1568 (11.2%) | 2811 (12.0%) |  |
| **Different Types of Albuminuria** |  |  |  |  |  | <0.001 |
| Non-Albuminuria | 5508 (88.2%) | 1608 (84.5%) | 1038 (83.0%) | 12486 (88.9%) | 20640 (88.0%) |  |
| Microalbuminuria | 605 (9.7%) | 234 (12.3%) | 177 (14.2%) | 1295 (9.2%) | 2311 (9.9%) |  |
| Macroalbuminuria | 130 (2.1%) | 62 (3.3%) | 35 (2.8%) | 271 (1.9%) | 498 (2.1%) |  |
