# Supplementary material for: Albuminuria and Mental Illness Risk: Results From National Health and Nutrition Examination Survey 2005–2018 and Mendelian Randomization Analyses
Source: Brain Behav. 2025 May 11;15(5):e70545. doi: 10.1002/brb3.70545 (PMC12066806; doi:10.1002/brb3.70545)
Supplement: Supplementary file 13 — Supporting Information. [file BRB3-15-e70545-s005.docx]

**Table S1.** Baseline characteristics of the research population with different types of Depression

**Table S2.** The source and definition of exposure and outcome

**Table S3.** Inclusion of instrumental variables

**Table S4.** MR analysis

**Table S5.** Sensitivity analysis

**Table S6.** Reverse MR analysis
