# Supplementary material for: Albuminuria and Mental Illness Risk: Results From National Health and Nutrition Examination Survey 2005–2018 and Mendelian Randomization Analyses
Source: Brain Behav. 2025 May 11;15(5):e70545. doi: 10.1002/brb3.70545 (PMC12066806; doi:10.1002/brb3.70545)
Supplement: Supplementary file 14 — Supporting Information. [file BRB3-15-e70545-s003.docx]

**Supplementary Figure Legends:**

**Figure S1. Subgroup analysis for the association between Albuminuria and Depression.** Association between albuminuria and depression by full-adjusted weighted multivariate logistic regression models. **A**: Forest plots showing association between albuminuria and depression. **B**: Association between albuminuria and depression. The odds ratio (OR) was estimated using the full-adjusted weighted multivariate logistic regression models. The horizontal bars represent 95% confidence intervals (CI). **C**: The P for interaction represents the results of the interaction tests.

**Figure S2. Subgroup analysis for the association between different types of Albuminuria and Depression.** Association between different types of albuminuria and depression by full-adjusted weighted multivariate logistic regression models. Association between different types of albuminuria and depression by full-adjusted weighted multivariate logistic regression models. **A**: Forest plots showing association between different types of albuminuria and depression. **B**: Association between different types of albuminuria and depression. The odds ratio (OR) was estimated using the full-adjusted weighted multivariate logistic regression models. The horizontal bars represent 95% confidence intervals (CI). **C**: The P for interaction represents the results of the interaction tests.

**Figure S3. Subgroup analysis for the association between UACR and Depression** Association between Log2-Transformed UACR (mg/g) and depression by full-adjusted weighted multivariate logistic regression models. Association between Log2-Transformed UACR and depression by full-adjusted weighted multivariate logistic regression models. **A**: Forest plots showing association between Log2-Transformed UACR and depression. **B**: Association between Log2-Transformed UACR and depression. The odds ratio (OR) was estimated using the full-adjusted weighted multivariate logistic regression models. The horizontal bars represent 95% confidence intervals (CI). **C** The P for interaction represent the results of the interaction tests.

**Figure S4.** **Scatter plots for MR analyses of the causal effect of albuminuria on mental illness.** The MR analyses were carried out utilizing various methods, including fixed-effect inverse variance weighting, weighted mean, MR-Egger, Weight mode, and Weight median. Each line's slope represents the estimated MR effect for the specific method, with error bars indicating the 95% confidence intervals around each SNP. **A:** anxiety disorder. **B:** persistent delusional disorder. **C:** depression. **D:** schizophrenia. **E:** schizotypal personality disorder. **F:** panic disorder. **G:** post-traumatic stress disorder. **H:** obsessive-compulsive disorder. **I:** bipolar I disorder. **J:** bipolar II disorder. **K:** social anxiety disorder. **L:** autism.

**Figure S5. Funnel plots for the causal effect of albuminuria on mental illness.**

Funnel plots were generated for Mendelian randomization (MR) analyses investigating the relationship between albuminuria and mental illness. These plots display the inverse variance weighted MR estimate for each albuminuria single-nucleotide polymorphism with cytokines plotted against 1/standard error (1/SEIV). **A:** anxiety disorder. **B:** persistent delusional disorder. **C:** depression. **D:** schizophrenia. **E:** schizotypal personality disorder. **F:** panic disorder. **G:** post-traumatic stress disorder. **H:** obsessive-compulsive disorder. **I:** bipolar I disorder. **J:** bipolar II disorder. **K:** social anxiety disorder. **L:** autism.

**Figure S6.** **Plots of leave-one-out analyses for the causal effect of albuminuria on mental illness.** Forest plots illustrating the causal estimates of albuminuria on mental illness by sequentially excluding each instrumental variable. The horizontal bars depict the beta value and its corresponding 95% confidence intervals (CI) for each causal estimate. **A:** anxiety disorder. **B:** persistent delusional disorder. **C:** depression. **D:** schizophrenia. **E:** schizotypal personality disorder. **F:** panic disorder. **G:** post-traumatic stress disorder. **H:** obsessive-compulsive disorder. **I:** bipolar I disorder. **J:** bipolar II disorder. **K:** social anxiety disorder. **L:** autism.
